# Supplementary figures and images for: Not all who wander are lost: Trail bias in community science
Source: PLoS One. 2023 Jun 23;18(6):e0287150. doi: 10.1371/journal.pone.0287150 (PMC10289309; doi:10.1371/journal.pone.0287150)

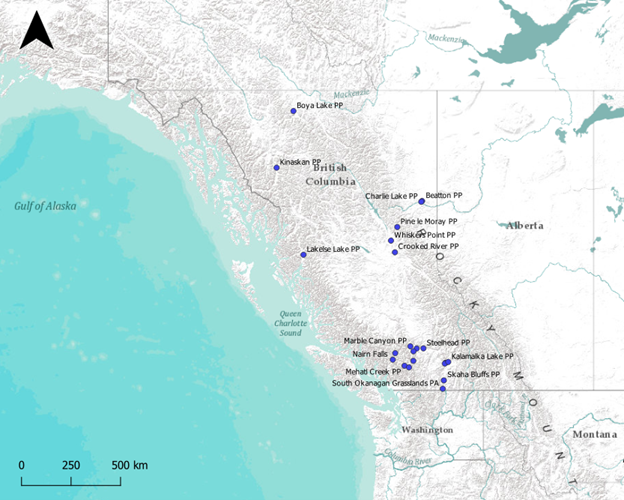

Supplement: S1 Fig — We used the ESRI Terrain and Reference Overlay basemaps from QGIS. (TIF) [file pone.0287150.s001.tif]
