# Supplementary material for: Not all who wander are lost: Trail bias in community science
Source: PLoS One. 2023 Jun 23;18(6):e0287150. doi: 10.1371/journal.pone.0287150 (PMC10289309; doi:10.1371/journal.pone.0287150)
Supplement: S1 Table — Dominant vegetation types are the tallest trees and shrubs with the most cover. PP = Provincial Park. PA = Protected Area. (PDF) [file pone.0287150.s002.pdf]

Park name	Number of paired transects	Dominant vegetation #1	Dominant vegetation #2	Dominant vegetation #3
**Beatton PP**	14	*Populus* sp.	*Picea* sp.	*Populus* sp. & *Picea* sp.
**Birkenhead Lake PP**	8	*Tsuga heterophylla*. & *Thuja plicata*	*Thuja heterophylla* & *Pseudotsuga menziesii*	*Acer macrophyllum*
**Boya Lake PP**	2	*Populus* sp.	*Populus* sp. & *Picea* sp.	-
**Charlie Lake PP**	8	*Populus* sp.	-	-
**Crooked River PP**	6	*Pinus contorta*	*Alnus* sp. & *Abies* sp.	*Pseudotsuga menziesii*
**Elephant Hill PP**	1	*Artemisia* sp.	-	-
**Ellison PP**	6	*Pseudotsuga menziesii* & *Pinus ponderosa*	*Pseudotsuga menziesii*	-
**Fintry PP**	4	*Populus* sp.	*Pseudotsuga menziesii* & *Pinus ponderosa*	*Medicago sativa*
**Kalamalka Lake PP**	8	*Symphoricarpos* sp. or *Amelanchier* sp.	*Pinus ponderosa*	*Pseudotsuga menziesii*
**Kinaskan PP**	3	*Picea* sp. & *Abies* sp.	*Picea* sp. & *Pinus contorta*	-
**Lakelse Lake PP**	3	*Tsuga heterophylla*	*Tsuga heterophylla* & *Populus* sp.	-
**Marble Canyon PP**	2	*Pseudotsuga menziesii*	-	-
**Mehatl Creek PP**	3	*Thuja plicata* & *Pseudotsuga menziesii*	*Pseudotsuga menziesii*	*Tsuga heterophylla*
**Nahatlatch PP**	3	*Pseudotsuga menziesii*	*Thuja* sp. & *Pseudotsuga menziesii*	-
**Nairn Falls PP**	4	*Thuja plicata* & *Tsuga heterophylla*	*Tsuga heterophylla*	*Thuja plicata*
**Oregon Jack PP**	4	*Pseudotsuga menziesii*	-	-
**Pine le Moray PP**	4	*Picea* sp.	*Picea* sp. & *Betula* sp.	*Pseudotsuga menziesii*
**Skaha Bluffs PP**	4	*Pinus ponderosa*	*Salix* sp.	*Amelanchier alnifolia* or *Philadelphus lewisii*
**Skihist PP**	4	*Pseudotsuga menziesii* & *Pinus ponderosa*	-	-
**South Okanagan Grasslands PA**	2	*Pseudotsuga menziesii*	*Pseudotsuga menziesii* & *Pinus ponderosa*	-
**Steelhead PP**	1	*Ericameria nauseosa*	-	-
**Whiskers Point PP**	2	*Pseudotsuga menziesii*	-	-
**Total**	**96**			
