# Supplementary material for: Not all who wander are lost: Trail bias in community science
Source: PLoS One. 2023 Jun 23;18(6):e0287150. doi: 10.1371/journal.pone.0287150 (PMC10289309; doi:10.1371/journal.pone.0287150)
Supplement: Attachment — Submitted filename: PONE-D-23-09371_reviewed.pdf [file pone.0287150.s003.pdf]

PLOS ONE

# Not all who wander are lost: trail bias in community science

--Manuscript Draft--

| | |
|---|---|
| **Manuscript Number:** | PONE-D-23-09371 |
| **Article Type:** | Research Article |
| **Full Title:** | Not all who wander are lost: trail bias in community science |
| **Short Title:** | Trail bias in community science |
| **Corresponding Author:** | Ellyne Geurts, B.Sc<br>University of Victoria Faculty of Social Sciences<br>Victoria, British Columbia CANADA |
| **Keywords:** | Biodiversity; Citizen science; Community science; iNaturalist; Opportunistic data; Parks; Presence-only; Sampling bias; Spatial bias; Taxonomic richness |
| **Abstract:** | The exponential growth and interest in community science programs is producing staggering amounts of biodiversity data across broad temporal and spatial scales. Large community science datasets such as iNaturalist and eBird are allowing ecologists and conservation biologists to answer novel questions that were not possible before. However, the opportunistic nature of many of these enormous datasets leads to biases. Spatial bias is a common problem, where observations are biased towards points of access like roads and trails. iNaturalist – a popular biodiversity community science platform – exhibits strong spatial biases, but it is unclear how these biases affect the quality of biodiversity data collected. Thus, we tested whether fine-scale spatial bias due to sampling from trails affects taxonomic richness estimates. We compared timed transects with experienced iNaturalist observers on and off trails in British Columbia, Canada. Using generalized linear mixed models, we found higher overall taxonomic richness on-trails than off-trails. In addition, we found more exotic as well as native taxa on-trails than off-trails. There was no difference between on and off trail observations for species that are rarely observed. Thus, fine-scale spatial bias from trails does not reduce the quality of biodiversity measurements, a promising result for those interested in using iNaturalist data for research and conservation management. |
| **Order of Authors:** | Ellyne Geurts |
| | John D Reynolds |
| | Brian M Starzomski |
| **Additional Information:** | |
| **Question** | **Response** |
| **Financial Disclosure**<br><br>Enter a financial disclosure statement that describes the sources of funding for the work included in this submission. Review the submission guidelines for detailed requirements. View published research articles from *PLOS ONE* for specific examples.<br><br>This statement is required for submission and **will appear in the published article** if the submission is accepted. Please make sure it is accurate. | This work was supported by BC Parks (https://bcparks.ca/ ), the BC Ministry of Forests, Land, Resource Operations and Rural Development (now known as the BC Ministry of Water, Land and Resource Stewardship: https://www2.gov.bc.ca/gov/content/governments/organizational-structure/ministries-organizations/ministries/water-land-and-resource-stewardship), the Sitka Foundation (https://sitkafoundation.org/), the Pacific Wildlife Foundation (https://pwlf.ca/), and the Natural Sciences and Engineering Research Council of Canada (NSERC: https://www.nserc-crsng.gc.ca/index_eng.asp). Provincial funding (BC Parks and the BC Ministry of Forests, Land, Resource Operations and Rural Development) was received by B.M.S. Funding from the Sitka Foundation (Grant #: 31-R831693) and the Pacific WildLife Foundation was received by J.D.R. In addition, NSERC Discovery grants were received by B.M.S. (Grant #: 04476) and J.D.R. (Grant #: 31-R611506). Lastly, a NSERC CGS-M scholarship was received by E.M.G.<br><br>The funders had no role in study design, data collection and analysis, decision to publish, or preparation of the manuscript. |

Powered by Editorial Manager® and ProduXion Manager® from Aries Systems Corporation

| | |
|---|---|
| **Unfunded studies**<br>Enter: *The author(s) received no specific funding for this work.*<br><br>**Funded studies**<br>Enter a statement with the following details:<br>• Initials of the authors who received each award<br>• Grant numbers awarded to each author<br>• The full name of each funder<br>• URL of each funder website<br>• Did the sponsors or funders play any role in the study design, data collection and analysis, decision to publish, or preparation of the manuscript?<br>• **NO** - Include this sentence at the end of your statement: *The funders had no role in study design, data collection and analysis, decision to publish, or preparation of the manuscript.*<br>• **YES** - Specify the role(s) played.<br><br>* typeset | |
| **Competing Interests**<br><br>Use the instructions below to enter a competing interest statement for this submission. On behalf of all authors, disclose any competing interests that could be perceived to bias this work—acknowledging all financial support and any other relevant financial or non-financial competing interests.<br><br>This statement is required for submission and **will appear in the published article** if the submission is accepted. Please make sure it is accurate and that any funding sources listed in your Funding Information later in the submission form are also declared in your Financial Disclosure statement.<br><br>View published research articles from *PLOS ONE* for specific examples. | The authors have declared that no competing interests exist. |

| | |
|---|---|
| **NO authors have competing interests**<br><br>Enter: *The authors have declared that no competing interests exist.*<br><br>**Authors with competing interests**<br><br>Enter competing interest details beginning with this statement:<br><br>*I have read the journal's policy and the authors of this manuscript have the following competing interests: [insert competing interests here]*<br><br>* typeset | |
| **Ethics Statement**<br><br>Enter an ethics statement for this submission. This statement is required if the study involved:<br><br>• Human participants<br>• Human specimens or tissue<br>• Vertebrate animals or cephalopods<br>• Vertebrate embryos or tissues<br>• Field research<br><br>Write "N/A" if the submission does not require an ethics statement.<br><br>General guidance is provided below. Consult the submission guidelines for detailed instructions. **Make sure that all information entered here is included in the Methods section of the manuscript.** | N/A |

Powered by Editorial Manager® and ProduXion Manager® from Aries Systems Corporation

| | |
|---|---|
| **Format for specific study types**<br><br>**Human Subject Research (involving human participants and/or tissue)**<br>• Give the name of the institutional review board or ethics committee that approved the study<br>• Include the approval number and/or a statement indicating approval of this research<br>• Indicate the form of consent obtained (written/oral) or the reason that consent was not obtained (e.g. the data were analyzed anonymously)<br><br>**Animal Research (involving vertebrate animals, embryos or tissues)**<br>• Provide the name of the Institutional Animal Care and Use Committee (IACUC) or other relevant ethics board that reviewed the study protocol, and indicate whether they approved this research or granted a formal waiver of ethical approval<br>• Include an approval number if one was obtained<br>• If the study involved *non-human primates*, add *additional details* about animal welfare and steps taken to ameliorate suffering<br>• If anesthesia, euthanasia, or any kind of animal sacrifice is part of the study, include briefly which substances and/or methods were applied<br><br>**Field Research**<br><br>Include the following details if this study involves the collection of plant, animal, or other materials from a natural setting:<br>• Field permit number<br>• Name of the institution or relevant body that granted permission | |
| **Data Availability**<br><br>Authors are required to make all data underlying the findings described fully available, without restriction, and from the time of publication. PLOS allows rare exceptions to address legal and ethical concerns. See the PLOS Data Policy and FAQ for detailed information. | Yes - all data are fully available without restriction |

Powered by Editorial Manager® and ProduXion Manager® from Aries Systems Corporation

| | |
|---|---|
| A Data Availability Statement describing where the data can be found is required at submission. Your answers to this question constitute the Data Availability Statement and **will be published in the article**, if accepted.<br><br>**Important:** Stating 'data available on request from the author' is not sufficient. If your data are only available upon request, select 'No' for the first question and explain your exceptional situation in the text box.<br><br>Do the authors confirm that all data underlying the findings described in their manuscript are fully available without restriction? | |
| **Describe where the data may be found in full sentences. If you are copying our sample text, replace any instances of XXX with the appropriate details.**<br><br>• If the data are **held or will be held in a public repository**, include URLs, accession numbers or DOIs. If this information will only be available after acceptance, indicate this by ticking the box below. For example: *All XXX files are available from the XXX database (accession number(s) XXX, XXX.).*<br>• If the data are all contained **within the manuscript and/or Supporting Information files**, enter the following: *All relevant data are within the manuscript and its Supporting Information files.*<br>• If neither of these applies but you are able to provide **details of access elsewhere**, with or without limitations, please do so. For example:<br><br>*Data cannot be shared publicly because of [XXX]. Data are available from the XXX Institutional Data Access / Ethics Committee (contact via XXX) for researchers who meet the criteria for access to confidential data.*<br><br>*The data underlying the results presented in the study are available from (include the name of the third party* | Data and code used in this study can be found on GitHub (https://github.com/ellynegeurts/Fine_scale_spatial_biases_iNaturalist_British_Columbia). This information can also be accessed on Zenodo (DOI: 10.5281/zenodo.7761835). |

| | |
|---|---|
| *and contact information or URL).*<br>• This text is appropriate if the data are owned by a third party and authors do not have permission to share the data.<br><br>* typeset | |
| Additional data availability information: | |

Powered by Editorial Manager® and ProduXion Manager® from Aries Systems Corporation

Manuscript
Click here to access/download;Manuscript;Geurts_Reynolds_Starzomski_PL

# Not all who wander are lost: trail bias in community science

Ellyne M. Geurts[1¶]*, John D. Reynolds[2¶], Brian M. Starzomski[1¶]*

[1] School of Environmental Studies, University of Victoria, 3800 Finnerty Rd., Victoria, British Columbia, Canada, V8P 5C2

[2] Earth to Ocean Research Group, Department of Biological Sciences, 8888 University Drive, Simon Fraser University, Burnaby, British Columbia, Canada, V5A 1S6

*Corresponding authors:

egeurts@uvic.ca (EMG)

starzom@uvic.ca (BMS)

[¶]These authors contributed equally to this work

# Abstract

The exponential growth and interest in community science programs is producing staggering amounts of biodiversity data across broad temporal and spatial scales. Large community science datasets such as iNaturalist and eBird are allowing ecologists and conservation biologists to answer novel questions that were not possible before. However, the opportunistic nature of many of these enormous datasets leads to biases. Spatial bias is a common problem, where observations are biased towards points of access like roads and trails. iNaturalist – a popular biodiversity community science platform – exhibits strong spatial biases, but it is unclear how these biases affect the quality of biodiversity data collected. Thus, we tested whether fine-scale spatial bias due to sampling from trails affects taxonomic richness estimates. We compared timed transects with experienced iNaturalist observers on and off trails in British Columbia, Canada. Using generalized linear mixed models, we found higher overall taxonomic richness on-trails than off-trails. In addition, we found more exotic as well as native taxa on-trails than off-trails. There was no difference between on and off trail observations for species that are rarely observed. Thus, fine-scale spatial bias from trails does not reduce the quality of biodiversity measurements, a promising result for those interested in using iNaturalist data for research and conservation management.


# Introduction

Community science, also called citizen science, involves data collected in collaboration between community members and scientists. It is growing rapidly in popularity in biodiversity research and management [1–3]. The advancements of technology and accessible platforms

like eBird and iNaturalist have increased participation and are producing massive amounts of biodiversity and ecological data around the world [4,5]. These data have been used to answer questions of phenology [6,7], species range changes [8], species migrations [9], phenotypic variation [10,11], species interactions [12], and body condition and disease [13]. The data from these platforms are also helping researchers discover new species and track invasive species [14,15], and improve species distribution models [16,17]. In addition to contributing large amounts of ecological data, community science platforms are educational resources that connect the public with their environments and with other like-minded individuals, cultivating a community of naturalists and environmental stewards [1,18,19]. As a result, researchers and environmental managers are keen to promote these platforms to collect more and better data.

iNaturalist engages the largest number of people globally of any biodiversity community science platform, with more than 125 million observations of taxa around the world, serving as a huge voucher photo and audio data repository [3]. The photo vouchers provide primary data on species occurrences, but also valuable secondary data such as life stage and sex, species interactions, and body condition. Users of iNaturalist are self-directed (*i.e.,* no sampling guidelines) and can make a species observation anywhere in the wor[illegible]his open-geographic feature allows users to contribute data across broad geographic scales that could never be obtained from traditional scientific surveys. Nevertheless, reservations remain regarding the quality of the data from iNaturalist due to its lack of sampling guidelines, which may lead to spatial, temporal, and taxonomic biases (Di Cecco et al., 2021).

Virtually all biodiversity datasets suffer from biases on spatial, temporal, taxonomic, and observer levels [21–25]. Community science data are frequently biased spatially by level of

accessibility such as proximity to roads and trails [26–28]. Observations are also biased towards tourist locations such as parks and protected areas [29,30] and by human population density [23,25,31]. These biases need to be understood and properly accounted for [32–34]. Some observational studies of the spatial, taxonomic, observer, and temporal biases in iNaturalist data have been conducted [20], but no experimental studies of iNaturalist biases and their impacts on biodiversity records have been conducted.

To address this gap, we tested fine-scale spatial impacts on taxonomic richness estimates. We compared observations made along trails and away from trails, because in many terrestrial habitats observations are biased toward trails due to accessibility [26,35,36]. This bias may be reinforced by rules in parks, where people are not allowed to leave trails [37]. We asked the question "Does trail spatial bias affect the number of taxa observed in parks by community scientists?". We conducted timed field experiments using experienced iNaturalist observers to compare taxonomic richness estimates between on-trail versus off-trail observations in provincial parks in British Columbia, Canada. The experiments emulated how an iNaturalist user would observe their environment with no distance or taxonomic restriction for each transect. We implemented a duration limit to aid comparability between the two treatments. We predicted higher taxonomic richness estimates on-trail compared to off-trail [38,39]. In addition, we predicted the species compositions to vary between on and off trail, with more exotic species observed on-trails [40]. Lastly, we examined differences in rare and vulnerable species observations between on and off trail.

# Materials and methods

## Study area and data

Sampling took place in 22 provincial parks and protected areas in British Columbia, Canada (S1 Fig). We conducted transects in three habitat types: grasslands, open-canopy forests, and closed-canopy forests. Figure 1 ~~shows example habitats and S1 Table shows dominant vegetation observed~~. Grasslands lack trees, open-canopy forests are dominated by pine trees (*Pinus* sp.), and closed-canopy forests are dominated by non-pine trees. ~~The full list of parks and habitats surveyed can be found in S1 Table.~~ We surveyed a variety of trail substrate types: bare ground (n = 63), gravel (n = 33), and asphalt (n = 1). Trail widths ranged from 0.5 m to 10 m with a mean of 3.1 m. Note that the 10 m trail was a groomed cross-country trail.

**Fig 1. Examples of habitats surveyed in British Columbia, Canada.** a) Closed-canopy forest (Beatton Provincial Park). **b)** Closed-canopy forest (Nairn Falls Provincial Park). **c)** Grassland (Steelhead Provincial Park). **d)** Open-canopy forest (Ellison Provincial Park). Photos taken by Kate McKeown (a-c) and Ellyne Geurts (d).

We defined taxonomic richness as the number of unique taxa per transect. This allowed for higher classification level observations to be included, as not all observations (*e.g.,* spiders and lichens) could be identified to species level by photographs alone [41,42]. We analyzed the

number of exotic, vulnerable, and rare species detected on and off trails. Exotic species are species that are established outside of their native range via anthropogenic means. The list of ~~exotic~~ species ~~(*i.e.,*~~ not-native to British Columbia~~)~~ was downloaded from the BC Species & Ecosystems Explorer from the Conservation Data Centre [43]. Vulnerable species have a status in British Columbia that ranges from "special concern" (S3) to "historical species or possibly extirpated communities" (SH). The full list can be found on the "BC rarities project" on iNaturalist.ca (https://inaturalist.ca/projects/bc-rarities?tab=about). We define rare species as species that have only one record on iNaturalist in the park where the transects were done. We downloaded the transect data from iNaturalist using its CSV Export Function on November 18, 2021 (n = 9,931 observations), and downloaded all observations recorded in the 22 parks on January 30, 2023 (n = 94,777 observations).

## iNaturalist

iNaturalist is a crowd-source type community science platform that was founded in 2008 and has since grown to over 2.5 million users globally [44]. It is hosted by the California Academy of Sciences and National Geographic Society. Users can upload georeferenced photo and audio recordings of any wild organism (*i.e.,* a verifiable observation). Photo identification can be aided by iNaturalist's computer vision program as well as input from the iNaturalist community. If the verifiable observation reaches majority agreement in species identification from the community, the quality grade of the observation is upgraded from "Needs ID" to "Research Grade", and depending on license restrictions by the observer, the data are

incorporated in the Global Biodiversity Information Facility (https://www.inaturalist.org/pages/help). iNaturalist produces opportunistic presence-only data.

## Field experiments

We conducted field experiments of trail spatial biases with a team of six iNaturalist observers from the BC Parks iNaturalist Program (BC iNaturalist Program, 2021; https://inaturalist.ca/projects/bc-parks and https://inaturalist.ca/projects/bc-parks-inat-team-big-summer-2021). The observers were active users with at least 3,500+ observations on iNaturalist and had prior experience surveying biodiversity in British Columbia. Observers did surveys together for the first two weeks with professional naturalists before beginning the field experiments to help standardize identification and search patterns. Data collection took place between May and August 2021 (S1 Table). We conducted 96 paired transects. We photographed the habitat at the beginning and end of each transect to help refine habitat categories. We conducted the paired transects in teams of three, with two observers and one facilitator for efficient data recording. One observer walked on the trail while the other observer walked parallel to the trail approximately 20 m away within the same habitat. We selected 20 m because trail effects from disturbance generally fade after 5 m away from the trail [46,47]. The facilitator followed behind the observer pair to keep time, gather all metadata for each transect, and ensure the off-trail observer stayed at least 20 m from the trail. The observer pairs photographed all plants, animals, and fungi that they encountered for 30 minutes in each transect. They were instructed simply to try to maximize the number of species

that they photographed. They recorded their straight-line distances using Garmin GPS units (*etrex 20*) as a proxy for distance traveled. We did not measure true distance traveled due to GPS signal interferences in forests and ravines resulting in unreliable GPS track distances. Transects did not have set distances to better emulate a typical iNaturalist user but we considered the effects of distances traveled in the statistics. The on-trail transects included a 2 m buffer on either side of the trail to mimic typical iNaturalist user behaviour in photographing species from trails. We selected trail segments that did not contain switchbacks to allow parallel surveying without backtracking. The side of the off-trail survey was selected randomly except when there were safety concerns.

During the on and off trail transects, the facilitator recorded trail width and type, and the dominant habitat type and mesohabitats that the observers encountered [48,49]. Mesohabitats included: cliff faces (large vertical rock), seeps (damp depressions), streams (narrow flowing water), and water pools (small areas with standing water). The observers employed the floristic habitat sampling method also referred to as the “intelligent meander” [50], where observers seek different mesohabitats and microhabitats (*e.g.,* logs and rocks) during transects. This sampling method observes more species, especially rarer species, than the traditional plot sampling method and is thought to more closely resemble the behaviour of iNaturalist users [48]. To ensure a balanced sampling design, each observer surveyed on and off trail an equal number of times throughout the summer. In addition, observer pairs switched regularly. Each observer surveyed with each partner the same number of times. To avoid temporal clustering of sampling for both trail position and observer partner, the rotations were

spaced out evenly throughout the summer. Surveys were done opportunistically throughout the summer when parks with adequate trails were available and weather permitted.

## Statistical analyses

We compared on versus off trail taxonomic richness using generalized linear mixed models with a Poisson distribution using the “glmer” function from the *lme4* R package [51]. Taxonomic richness is defined as the number of unique taxa recorded per transect. We included observer identity as a random effect and the trail variable of on- and off- position as a fixed effect. We also included location of transect as a random effect with trail name nested within park name. In addition, we considered habitat type, number of observations, and distance traveled as additional explanatory variables. We standardized the distance traveled variable to have a mean of zero and standard deviation of one. We excluded the variable of mesohabitats encountered as most of the transects encountered zero mesohabitats (n = 168 out of 192 individual transects), meaning there was only one dominant habitat type throughout the transect. We used random intercepts for the variance structure. We did not test for random slopes for different observers due to high ratio of parameters to sample size. We compared four models with different fixed-effect structures using AICc: (1) a full model with trail position (on vs. off), habitat type, and distance traveled, (2) a full model with an interaction term for distance traveled and trail position, as distance traveled could be affected by whether the observer is on or off trail, (3) a model with only the trail position variable, and (4) a null model.

We then repeated these steps with exotic species observations removed from the analysis (n = 391 observations removed) to compare native taxonomic richness.

We compared the number of rare species observed on or off trails using a Wilcoxon sign rank test. We began by filtering all observations in each park to only contain species-level observations (n = 57,843), then we removed all “casual” quality grade observations from the dataset (n = 57,547). For each park we created individualized rare species lists by selecting species that only had one record in the park. This allowed us to count the number of rare species observations recorded by us on and off trail by park. We treated each park as a pair (n = 22). We also counted the number of vulnerable species recorded on and off trails across all parks. Similarly, we compared the number of exotic species observed between on and off trail using a Wilcoxon sign rank test. We grouped transects by park and summed the number of exotic species observations recorded on and off trail. We also calculated the proportion of observations in each transect that contained exotic species.

We conducted the statistical analyses in R (version 4.1.2.) and RStudio [52,53]. The packages we used were *lme4* [51], *lubridate* [54], *dplyr* [55], *bbmle* [56], *sp* [57], *rgdal* [58], *adehabitatLT* [59], and *ggplot2* [60]. We archived the data and statistical analysis code we used on Zenodo (DOI: 10.5281/zenodo.7761835).

# Results

## Trail bias – taxonomic richness estimates

The observers recorded a total of 9,931 observations (on-trail = 5,107, off-trail = 4,824) of 1,323 taxa across the 96 paired transects. As predicted, there was higher taxonomic richness on-trails than off-trails. The mean number of taxa on-trail was 40.8, while off-trail was 37.6.

The top model was the full model with all variables included and no interaction between trail position and distance traveled (Table 1). Both the trail position and habitat variables were significant in that model (Fig 2). The grassland habitat had a lower number of observed taxa than the closed-canopy forest habitat (Figs 2 and 3). The number of taxa observed was highly correlated with number of observations (Pearson's product-moment correlation: $r = 0.90$, $t = 29.27$, $df = 190$, $p < 0.001$). Distance traveled did not affect the number of taxa observed (Fig 2). Mean and standard error of distance traveled by observers on-trail was 114 ± 9 m and 89 ± 6 m for off-trail.

**Fig 2. Standardized coefficient estimates with standard error bars for variables in the top model examining taxonomic richness by trail position, habitat type, and straight-line distance traveled by observer**. Trail position is "on" or "off". The three habitat types were "grassland", "open-canopy forest" and "closed-canopy forest". * = $p < 0.05$, ** = $p < 0.01$, and *** = $p < 0.001$. Blue indicates a negative relationship between the covariate and taxonomic richness,

while brown indicates a positive relationship. a) Total taxonomic richness model. b) Native taxonomic richness.

**Fig 3. ~~Boxplot of taxonomic richness~~ observed per transect across the three habitat types.** Taxonomic richness is the number of unique taxa on iNaturalist per transect. Black bar = median. Box = interquartile range. See Fig 2 for the model coefficient results.

**Table 1. Models tested to explain taxonomic richness observed along transects.**

| Model name | Variables tested | ΔAICc | DF | Weight |
|---|---|---|---|---|
| ***<u>Total taxonomic richness</u>*** | | | | |
| **Full model** | Trail position + distance + habitat | 0 | 8 | 0.671 |
| **Full model with distance interaction** | Trail position + distance + habitat + trail position:distance | 2.1 | 9 | 0.231 |
| **Trail position only** | Trail position | 3.9 | 5 | 0.097 |
| **Null model** | 1 | 15.1 | 4 | < 0.001 |
| ***<u>Native taxonomic richness</u>*** | | | | |
| **Full model** | Trail position + distance + habitat | 0 | 8 | 0.729 |
| **Full model with distance interaction** | Trail position + distance + habitat + trail position:distance | 2.1 | 9 | 0.256 |
| **Trail position only** | Trail position | 8.3 | 5 | 0.012 |
| **Null model** | 1 | 10.4 | 4 | 0.004 |

When we restricted the analysis to native taxa, the same top model explained taxonomic richness (Table 1). The pattern of variable importance is also similar, except 'open-canopy forest' now has an effect (Fig 2).

Total taxonomic richness is the total number of unique taxa observed per transect. Native taxonomic richness is total taxonomic richness with exotic species removed (n = 391 removed). Trail position indicates whether the observer was on or off trail. Distance = straight-line distance traveled during a transect. Habitat is classified into three broad categories reflecting general vegetation structure. All models had observer and trail name nested within park name as random effects.

## Trail bias - vulnerable, rare, and exotic species

Seven of the 192 individual transects contained at least one vulnerable species (on-trail = 6 transects, off-trail = 1 transect). Only one transect had more than one vulnerable species (n = 2). Examples of vulnerable species are shown in Fig 4. We observed four vulnerable insect species: Bunch Grass Locust (*Pseudopomala brachyptera*), a potter wasp (*Odynerus dilectus*), Kiowa Grasshopper (*Trachyrhachys kiowa*), and Huron Short-winged Locust (*Melanoplus huroni*). We also observed one bird species (White-throated Swift - *Aeronautes saxatalis*) and one plant species (Large-flowered Triteleia (*Triteleia grandiflora*). There was no difference in number of rare species between on and off trail (Wilcoxon signed rank test with continuity correction: V = 73.5, p = 0.40).

**Fig 4. Vulnerable species observed during transects on-trail in British Columbia, Canada.** Vulnerable species range from "special concern" (S3) to "historical species or possibly extirpated communities" (SH) in British Columbia. **a)** Bunch Grass Locust (*Pseudopomala brachyptera*) observed in Steelhead Provincial Park. **b)** *Odynerus dilectus* observed in Fintry Provincial Park and Protected Area. **c)** Kiowa Grasshopper (*Trachyrhachys kiowa*) observed in Steelhead Provincial Park. **d)** Large-flowered Triteleia (*Triteleia grandiflora*) observed in Kalamalka Lake Provincial Park. Photos taken by Lena Dietz Chiasson (a-c) and Erin Springinotic (d).

For exotic species, 110 of the 192 individual transects had at least one exotic species (Table 2). As predicted, we observed more exotic species on than off trail (Wilcoxon signed rank test with continuity correction: V = 2.5, $p < 0.001$). Exotic species appeared to make up a higher proportion of the observations for transects in grassland habitats than in forest habitats (Fig 5). See Table 2 for mean and range of number of exotic species observed on and off trails.

**Table 2. Summary statistics for the number of exotic observations and species recorded by trail position for all transects (n = 96 paired transects).**

| Trail position | Number of transects that encountered at least one exotic species | Mean number of exotic observations | Range of number of exotic observations | Mean number of exotic species | Range of number of exotic species |
|---|---|---|---|---|---|
| Off | 38 | 1.10 | 0 - 15 | 0.89 | 0 - 11 |
| On | 72 | 2.97 | 0 - 15 | 2.43 | 0 - 10 |

**Fig 5. ~~Boxplot of~~ the proportion of exotic species observed per transect across the three habitat types.** Proportion is calculated out of the total taxonomic richness observed per transect. Black bar = median. Box = interquartile range.

## Discussion

Higher taxonomic richness observed on-trails than off-trails during our 30-minute biodiversity transects suggests that fine-scale bias (*i.e.,* trail bias) does not lead to reduced biodiversity measurements from opportunistic community science. The lack of difference between on and off trail transects for rare (*i.e.,* uncommonly recorded) species also supports the inference that trail bias does not limit the quality of biodiversity observations derived strictly from trails. These results are encouraging evidence for park managers and conservation researchers: they show that spatially biased data from opportunistic community science can provide comparable or stronger taxonomic richness estimates on-trails compared to off-trails in a variety of habitats.

### Trail bias - taxonomic richness

Our test for effects of trails on estimates of taxonomic richness is novel because it includes all taxa in a variety of habitats in time-controlled transects where observers mimicked typical behaviour of people using community science. Previous results using standardized

surveys have been mixed regarding trail impacts on species diversity [61]. Some studies have found higher species richness on-trails for vascular plants and ground-dwelling arthropods [38,39,46,62], while other studies found no difference *e.g.* plants [63] or even higher species richness away from trails *e.g.,* lichens, flower-visiting insects, and gall-inducing insects [63–66].

There are a few potential explanations for why we observed higher taxonomic richness on-trail than off-trail. It is possible that there were indeed more species on-trails, perhaps because of more light and disturbance, which could favour ruderal species [39,61,62]. However, species may also have been more readily detected from trails due to increased visibility and greater ease of observers moving through the habitat. As the number of taxa observed was highly correlated with number of observations, it is likely that observing from trails imparts advantages for biodiversity surveying, allowing for more species to be recorded than off-trail. Either way, if people want to see as many species as possible (and most naturalists do), the message is that they will do so by following trail etiquette.

## Trail bias - species composition differences

Our finding of more exotic species along trails than off-trails agrees with other studies [40,67] and provides reassuring evidence for potential rapid detection of new invasives in regions [15,68]. This result might misleadingly give the impression that the higher taxonomic richness on-trails versus off-trails is due to exotic species locally boosting trailside taxonomic richness. However, when we removed exotic species from the models, we still found evidence of increased taxonomic richness of native taxa on trails.

The ~~lack of difference~~ in detection of rare species on versus off trails also suggests that observing from trails does not impose a disadvantage on biodiversity measurements. In addition, we found more vulnerable species along trails than off-trails, though our sample sizes were too small to make strong inferences. ~~It is interesting to note that~~ most of the vulnerable species that we observed were winged *i.e.,* mobile species (Fig 4). It is thus possible that vulnerable plants and lichens could be less commonly observed on-trail and consequently be underrepresented on iNaturalist [63].

In summary, our finding of higher taxonomic richness observed along trails than away from trails by iNaturalist observers is a promising outcome for the value of the platform for surveying biodiversity. Of course, there are still going to be strong biases due to the lack of sampling guidelines on iNaturalist, and spatial coverage will be imperfect. For example, some microhabitats (*e.g.,* logs, rocks, stumps) and mesohabitats (*e.g.,* seeps, cliffs, wetlands) and their inhabitants may be underrepresented by observers who stay on trails. Targeted surveys would be needed to complement community science for species in such places. In addition, the higher number of exotic species documented along trails and lack of difference detected for rare species supports the value of these data for tracking invasive species and for locating places where rare and vulnerable species occur. We hope that the results of more experiments like ours can further illuminate biases and enhance our ability to use the data effectively.

# Acknowledgements

We want to give a huge thank you to the field crew: Jason Headley, Erin Springinotic, Tori Miller, Kate McKeown, and Lena Dietz Chiasson for all their hard work and awesome observations. We also appreciate the 2,620 iNaturalist identifiers who volunteered their time and expertise to identify the observations we uploaded. The top 10 identifiers were Doug Brown (@dougbrown), Barbara L. Wilson (@sedgequeen), Randal Mindell (@rambryum), Finn McGhee (@fmcghee), Steve Ansell (@steveansell), Stephen B. Brown (@sbbrown), Jason Headley (@jasonheadley), Liam Steele (@pacificwhitesideddolphin), Stewart Wechsler (@stewartwechsler), and Peter L. Achuff (@plachuff)( https://inaturalist.ca/projects/bc-parks-inat-team-big-summer-2021?tab=identifiers). In addition, we appreciate the support from other members of the Reynolds and Starzomski labs for their creativity, input, and statistical advice on this project.

# References

1. Pocock MJO, Chandler M, Bonney R, Thornhill I, Albin A, August T, et al. A Vision for Global Biodiversity Monitoring With Citizen Science. 1st ed. Advances in Ecological Research. Elsevier Ltd.; 2018. doi:10.1016/bs.aecr.2018.06.003

2. Miller-Rushing A, Primack R, Bonney R. The history of public participation in ecological research. Front Ecol Environ. 2012;10: 285–290. doi:10.1890/110278

3. Loarie S. We’ve passed 100,000,000 verifiable observations on iNaturalist! In: iNaturalist

[Internet]. 2022 [cited 24 Aug 2022]. Available: https://www.inaturalist.org/blog/66531-we-ve-passed-100-000-000-verifiable-observations-on-inaturalist

4. Sullivan BL, Wood CL, Iliff MJ, Bonney RE, Fink D, Kelling S. eBird: a citizen-based bird observation network in the biological sciences. Biol Conserv. 2009;142: 2282–2292. doi:10.1016/j.biocon.2009.05.006

5. Loarie S. We've reached 1,000,000 observers! · iNaturalist. In: iNaturalist [Internet]. 2020 [cited 10 Mar 2021]. Available: https://www.inaturalist.org/blog/35758-we-ve-reached-1-000-000-observers

6. Barve V V., Brenskelle L, Li D, Stucky BJ, Barve N V., Hantak MM, et al. Methods for broad-scale plant phenology assessments using citizen scientists' photographs. Appl Plant Sci. 2020;8: 1–10. doi:10.1002/aps3.11315

7. Nowak K, Berger J, Panikowski A, Reid DG, Jacob AL, Newman G, et al. Using community photography to investigate phenology: A case study of coat molt in the mountain goat (*Oreamnos americanus*) with missing data. Ecol Evol. 2020;10: 13488–13499. doi:10.1002/ece3.6954

8. Johnston A, Hochachka WM, Strimas-Mackey ME, Ruiz Gutierrez V, Robinson OJ, Miller ET, et al. Analytical guidelines to increase the value of community science data: An example using eBird data to estimate species distributions. Divers Distrib. 2021;27: 1265–1277. doi:10.1111/ddi.13271

9. Walker J, Taylor PD. Using eBird data to model population change of migratory bird

species. Avian Conserv Ecol. 2017;12. doi:10.5751/ace-00960-120104

10. Drury JP, Barnes M, Finneran AE, Harris M, Grether GF. Continent-scale phenotype mapping using citizen scientists' photographs. Ecography (Cop). 2019;42: 1436–1445. doi:10.1111/ecog.04469

11. Lehtinen RM, Carlson BM, Hamm AR, Riley AG, Mullin MM, Gray WJ. Dispatches from the neighborhood watch: Using citizen science and field survey data to document color morph frequency in space and time. Ecol Evol. 2020;10: 1526–1538. doi:10.1002/ece3.6006

12. Saldivar JA, Romero AN, Wilson Rankin EE. Community science reveals high diversity of nectaring plants visited by painted lady butterflies (Lepidoptera: Nymphalidae) in California sage scrub. Environ Entomol. 2022; 1–9. doi:10.1093/ee/nvac073

13. Hamilton SL, Saccomanno VR, Heady WN, Gehman AL, Lonhart SI, Beas-Luna R, et al. Disease-driven mass mortality event leads to widespread extirpation and variable recovery potential of a marine predator across the eastern Pacific. Proceedings of the Royal Society B: Biological Sciences. 2021. doi:10.1098/rspb.2021.1195

14. Bois ST, Silander JA, Mehrhoff LJ. Invasive plant atlas of new England: The role of citizens in the science of invasive alien species detection. Bioscience. 2011;61: 763–770. doi:10.1525/bio.2011.61.10.6

15. Werenkraut V, Baudino F, Roy HE. Citizen science reveals the distribution of the invasive harlequin ladybird (Harmonia axyridis Pallas) in Argentina. Biol Invasions. 2020;22: 2915–

2921. doi:10.1007/s10530-020-02312-7

16. Matutini F, Baudry J, Pain G, Sineau M, Pithon J. How citizen science could improve species distribution models and their independent assessment. Ecol Evol. 2021;11: 3028–3039. doi:10.1002/ece3.7210

17. Feldman MJ, Imbeau L, Marchand P, Mazerolle MJ, Darveau M, Fenton NJ. Trends and gaps in the use of citizen science derived data as input for species distribution models: A quantitative review. PLoS One. 2021;16: 1–21. doi:10.1371/journal.pone.0234587

18. Kobori H, Dickinson JL, Washitani I, Sakurai R, Amano T, Komatsu N, et al. Citizen science: a new approach to advance ecology, education, and conservation. Ecol Res. 2016;31: 1–19. doi:10.1007/s11284-015-1314-y

19. Dickinson JL, Shirk J, Bonter D, Bonney R, Crain RL, Martin J, et al. The current state of citizen science as a tool for ecological research and public engagement. Front Ecol Environ. 2012;10: 291–297. doi:10.1890/110236

20. Di Cecco GJ, Barve V, Belitz MW, Stucky BJ, Guralnick RP, Hurlbert AH. Observing the observers: how participants contribute data to iNaturalist and implications for biodiversity science. Bioscience. 2021;71: 1179–1188. doi:10.1093/biosci/biab093

21. Troudet J, Grandcolas P, Blin A, Vignes-Lebbe R, Legendre F. Taxonomic bias in biodiversity data and societal preferences. Sci Rep. 2017;7: 1–14. doi:10.1038/s41598-017-09084-6

22. Isaac NJB, Pocock MJO. Bias and information in biological records. Biol J Linn Soc.

2015;115: 522–531. doi:10.1111/bij.12532

23. Speed JDM, Bendiksby M, Finstad AG, Hassel K, Kolstad AL, Prestø T. Contrasting spatial, temporal and environmental patterns in observation and specimen based species occurrence data. PLoS One. 2018;13: 1–17. doi:10.1371/journal.pone.0196417

24. Oliveira U, Paglia AP, Brescovit AD, de Carvalho CJB, Silva DP, Rezende DT, et al. The strong influence of collection bias on biodiversity knowledge shortfalls of Brazilian terrestrial biodiversity. Divers Distrib. 2016;22: 1232–1244. doi:10.1111/ddi.12489

25. Ruete A. Displaying bias in sampling effort of data accessed from biodiversity databases using ignorance maps. Biodivers Data J. 2015;3. doi:10.3897/BDJ.3.e5361

26. Geldmann J, Heilmann-Clausen J, Holm TE, Levinsky I, Markussen B, Olsen K, et al. What determines spatial bias in citizen science? Exploring four recording schemes with different proficiency requirements. Divers Distrib. 2016;22: 1139–1149. doi:10.1111/ddi.12477

27. El-Gabbas A, Dormann CF. Improved species-occurrence predictions in data-poor regions: using large-scale data and bias correction with down-weighted Poisson regression and Maxent. Ecography (Cop). 2018;41: 1161–1172. doi:10.1111/ecog.03149

28. Stolar J, Nielsen SE. Accounting for spatially biased sampling effort in presence-only species distribution modelling. Divers Distrib. 2015;21: 595–608. doi:10.1111/ddi.12279

29. Boakes EH, McGowan PJK, Fuller RA, Chang-Qing D, Clark NE, O'Connor K, et al. Distorted views of biodiversity: spatial and temporal bias in species occurrence data. PLoS Biol.

2010;8. doi:10.1371/journal.pbio.1000385

30. Rocchini D, Hortal J, Lengyel S, Lobo JM, Jiménez-Valverde A, Ricotta C, et al. Accounting for uncertainty when mapping species distributions: The need for maps of ignorance. Prog Phys Geogr. 2011;35: 211–226. doi:10.1177/0309133311399491

31. Ballesteros-Mejia L, Kitching IJ, Jetz W, Nagel P, Beck J. Mapping the biodiversity of tropical insects: species richness and inventory completeness of African sphingid moths. Glob Ecol Biogeogr. 2013;22: 586–595. doi:10.1111/geb.12039

32. Johnston A, Fink D, Hochachka WM, Kelling S. Estimates of observer expertise improve species distributions from citizen science data. Methods Ecol Evol. 2018;9: 88–97. doi:10.1111/2041-210X.12838

33. Fithian W, Elith J, Hastie T, Keith DA. Bias correction in species distribution models: Pooling survey and collection data for multiple species. Methods Ecol Evol. 2015;6: 424–438. doi:10.1111/2041-210X.12242

34. Courter JR, Johnson RJ, Stuyck CM, Lang BA, Kaiser EW. Weekend bias in citizen science data reporting: implications for phenology studies. Int J Biometeorol. 2013;57: 715–720. doi:10.1007/s00484-012-0598-7

35. Callaghan CT, Ozeroff I, Hitchcock C, Chandler M. Capitalizing on opportunistic citizen science data to monitor urban biodiversity: A multi-taxa framework. Biol Conserv. 2020;251: 108753. doi:10.1016/j.biocon.2020.108753

36. Jackson MM, Gergel SE, Martin K. Citizen science and field survey observations provide

comparable results for mapping Vancouver Island White-tailed Ptarmigan (Lagopus leucura saxatilis) distributions. Biol Conserv. 2015;181: 162–172. doi:10.1016/j.biocon.2014.11.010

37. Filazzola A, Xie G, Barrett K, Dunn A, Johnson MTJ, MacIvor JS. Using smartphone-GPS data to quantify human activity in green spaces. PLoS Comput Biol. 2022;18: e1010725. doi:10.1371/journal.pcbi.1010725

38. Wedegärtner REM, Lembrechts JJ, van der Wal R, Barros A, Chauvin A, Janssens I, et al. Hiking trails shift plant species' realized climatic niches and locally increase species richness. Divers Distrib. 2022;28: 1416–1429. doi:10.1111/ddi.13552

39. Root-Bernstein M, Svenning JC. Human paths have positive impacts on plant richness and diversity: A meta-analysis. Ecol Evol. 2018;8: 11111–11121. doi:10.1002/ece3.4578

40. Liedtke R, Barros A, Essl F, Lembrechts JJ, Wedegärtner REM, Pauchard A, et al. Hiking trails as conduits for the spread of non-native species in mountain areas. Biol Invasions. 2020;22: 1121–1134. doi:10.1007/s10530-019-02165-9

41. Mesaglio T, Callaghan CT, Samonte F, Gorta SBZ, Cornwell WK. Recognition and completeness: two key metrics for judging the utility of citizen science data. Front Ecol Environ. 2023; 1–8. doi:10.1002/fee.2604

42. McMullin RT, Allen JL. An assessment of data accuracy and best practice recommendations for observations of lichens and other taxonomically difficult taxa on iNaturalist. Botany. 2022;100: 491–497. doi:10.1139/cjb-2021-0160

43. B.C. Conservation Data Centre. BC Species and Ecosystems Explorer. In: B.C. Government, Victoria B.C. 2022.

44. iNaturalist. iNaturalist. 2022 [cited 11 May 2022]. Available: https://www.inaturalist.org

45. BC iNaturalist Program. BC iNaturalist. 2021 [cited 18 May 2022]. Available: https://www.bcinat.com/

46. Swart RC, Pryke JS, Roets F. The intermediate disturbance hypothesis explains arthropod beta-diversity responses to roads that cut through natural forests. Biol Conserv. 2019;236: 243–251. doi:10.1016/j.biocon.2019.03.045

47. Avon C, Bergès L, Dumas Y, Dupouey JL. Does the effect of forest roads extend a few meters or more into the adjacent forest? A study on understory plant diversity in managed oak stands. For Ecol Manage. 2010;259: 1546–1555. doi:10.1016/j.foreco.2010.01.031

48. Newmaster SG, Belland RJ, Arsenault A, Vitt DH, Stephens TR. The ones we left behind: comparing plot sampling and floristic habitat sampling for estimating bryophyte diversity. Divers Distrib. 2005;11: 57–72. doi:10.1111/j.1366-9516.2005.00123.x

49. McMullin RT, Wiersma YF. Lichens and allied fungi of Salmonier Nature Park, Newfoundland. J Torrey Bot Soc. 2017;144: 357–369. doi:10.3159/TORREY-D-16-00041

50. Selva SB. Using calicioid lichens and fungi to assess ecological continuity in the Acadian Forest Ecoregion of the Canadian Maritimes. For Chron. 2003;79: 550–558. doi:10.5558/tfc79550-3

51. Bates D, Maechler M, Bolker B, Walker S. Fitting Linear Mixed-Effects Models Using lme4. J Stat Softw. 2015;67: 1–48. doi:10.18637/jss.v067.i01

52. R Core Team. R: A language and environment for statistical computing. Vienna, Austria: R Foundation for Statistical Computing; 2021. Available: https://www.r-project.org/

53. RStudio Team. RStudio: integrated development environment for R. Boston, MA: RSudio, PBC; 2021. Available: http://www.rstudio.com/

54. Grolemund G, Wickham H. Dates and Times Made Easy with lubridate. J Stat Softw. 2011;40: 1–25. Available: https://www.jstatsoft.org/v40/i03/

55. Wickham H, François R, Henry L, Müller K. dplyr: a grammar of data manipulation. R package version 1.0.7. 2021. Available: https://cran.r-project.org/package=dplyr

56. Bolker B, R Development Core Team. bbmle: Tools for General Maximum Likelihood Estimation. R package version 1.0.25. 2022. Available: https://cran.r-project.org/package=bbmle

57. Pebesma EJ, Bivand RS. Classes and methods for spatial data in R. R News. 5: 9–13. Available: https://cran.r-project.org/doc/Rnews/

58. Bivand R, Keitt T, Rowlingson B. rgdal: Bindings for the “Geospatial” Data Abstraction Library. 2021. Available: https://cran.r-project.org/package=rgdal

59. Calenge C. The package adehabitat for the R software: tool for the analysis of space and habitat use by animals. Ecol Modell. 2006;197: 516–519.

60. Wickham H. ggplot2: Elegant Graphics for Data Analysis. New York: Springer-Verlag;

2016. Available: https://ggplot2.tidyverse.org

61. Ballantyne M, Pickering CM. The impacts of trail infrastructure on vegetation and soils: Current literature and future directions. J Environ Manage. 2015;164: 53–64. doi:10.1016/j.jenvman.2015.08.032

62. Queiroz RE, Ventura MA, Silva L. Plant diversity in hiking trails crossing Natura 2000 areas in the Azores: Implications for tourism and nature conservation. Biodivers Conserv. 2014;23: 1347–1365. doi:10.1007/s10531-014-0669-7

63. Jägerbrand AK, Alatalo JM. Effects of human trampling on abundance and diversity of vascular plants, bryophytes and lichens in alpine heath vegetation, Northern Sweden. Springerplus. 2015;4. doi:10.1186/s40064-015-0876-z

64. Kamel M. Impact of hiking trails on the diversity of flower-visiting insects in Wadi Telah, St. Katherine protectorate, Egypt. J Basic Appl Zool. 2020;81. doi:10.1186/s41936-020-00188-6

65. Kamel M. Hiking trails effects on the diversity of gall-inducing insects in high altitude ecosystem, St. Katherine Protectorate, Egypt. Zool Middle East. 2021;67: 48–56. doi:10.1080/09397140.2020.1859975

66. De Almeida ES, Sartori RA, Zaú AS. Trail Impacts in a Tropical Rainforest National Park. Geogr Environ Sustain. 2022;15: 5–12. doi:10.24057/2071-9388-2021-036

67. Ngugi MR, Neldner VJ, Dowling R. Non-native plant species richness adjacent to a horse trail network in seven National Parks in southeast Queensland, Australia. Australas J

Environ Manag. 2014;21: 413–428. doi:10.1080/14486563.2014.952788

68. Tiralongo F, Crocetta F, Riginella E, Lillo AO, Tondo E, Macali A, et al. Snapshot of rare, exotic and overlooked fish species in the Italian seas: A citizen science survey. J Sea Res. 2020;164: 101930. doi:10.1016/j.seares.2020.101930

# Supporting information

**S1 Fig. The 22 provincial parks and protected areas where we conducted transects between May and August 2021, in British Columbia, Canada.** We used the ESRI Terrain and Reference Overlay basemaps from QGIS.

**S1 Table. Provincial parks and protected areas visited along with the number of paired transects conducted and the dominant vegetation encountered for each site.** Dominant vegetation types are the tallest trees and shrubs with the most cover. PP = Provincial Park. PA = Protected Area.

Figure 1

Click here to access/download;Figure;Fig1.tiff

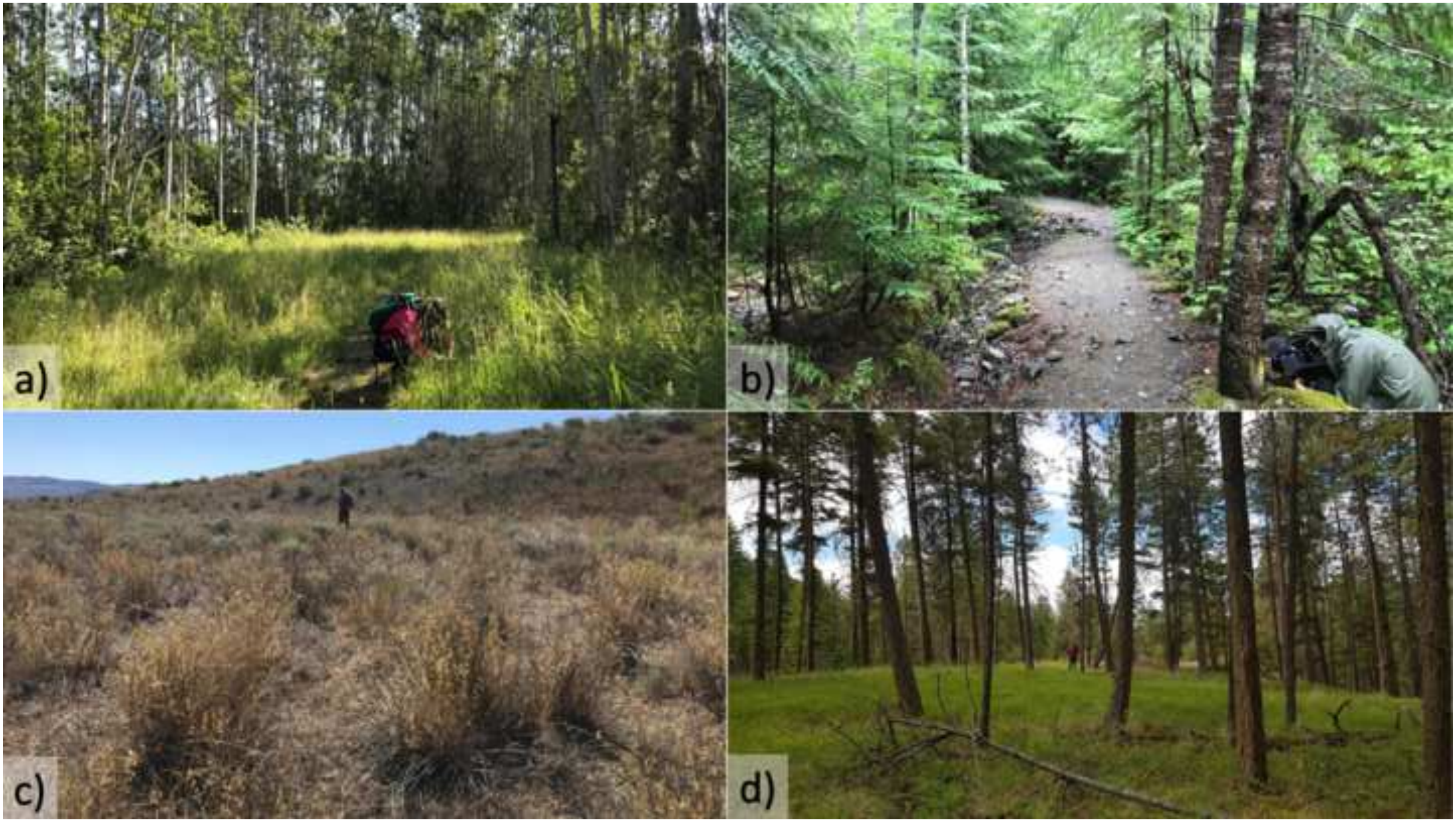

Figure 2

Click here to access/download;Figure;Fig2.tif

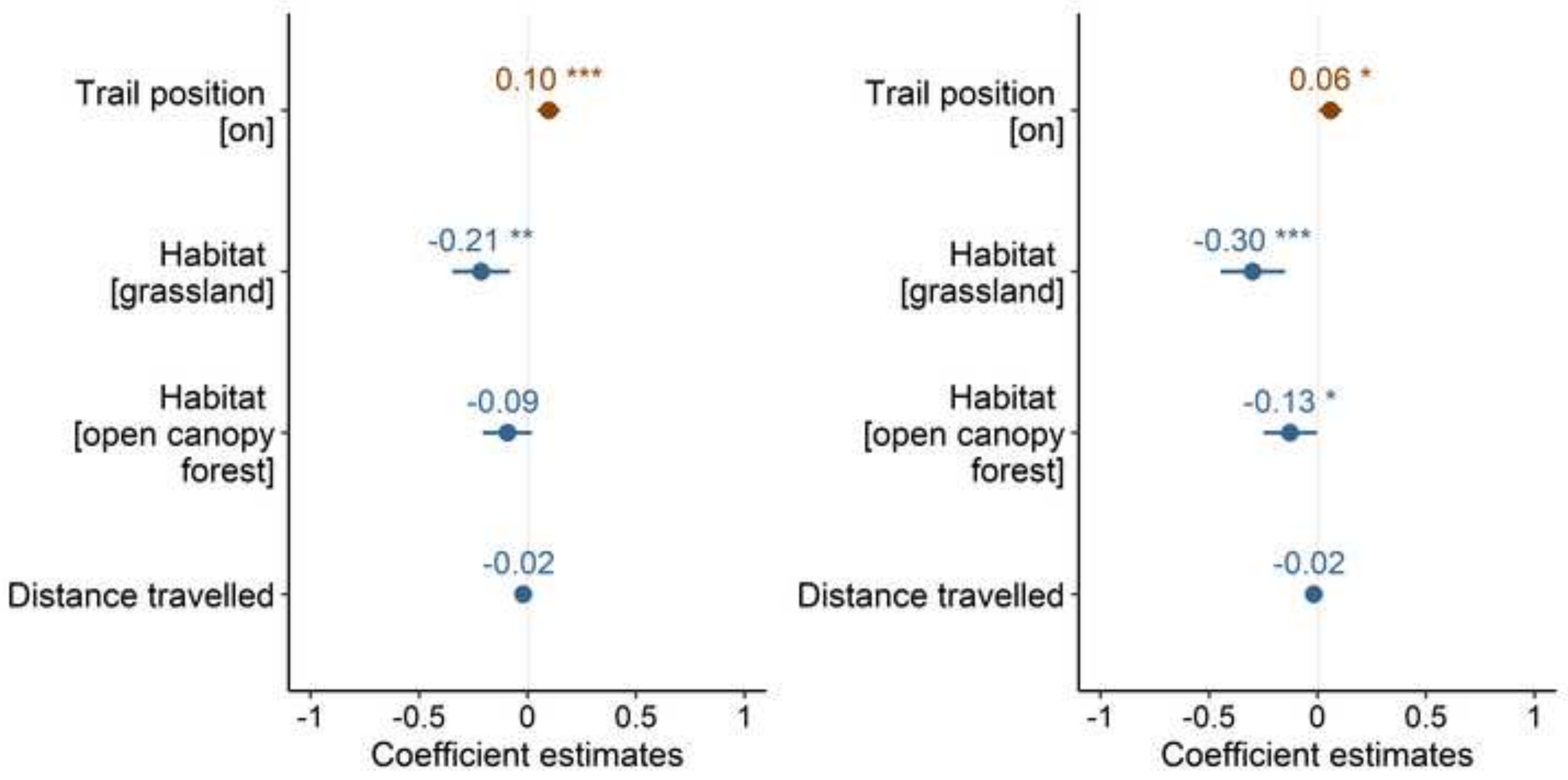

Figure 3

Click here to access/download;Figure;Fig3.tif

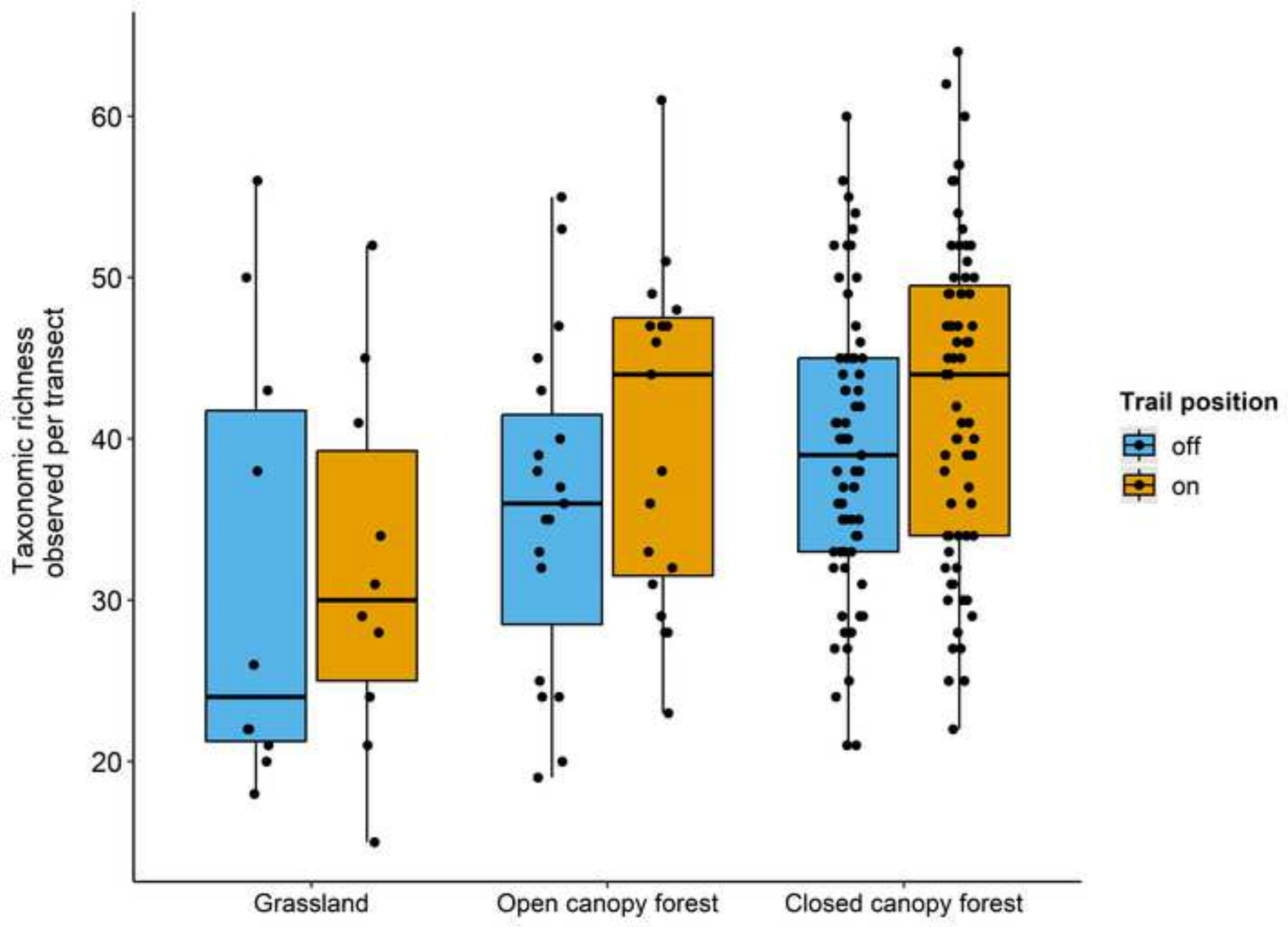

Figure 4

Click here to access/download;Figure;Fig4.tiff

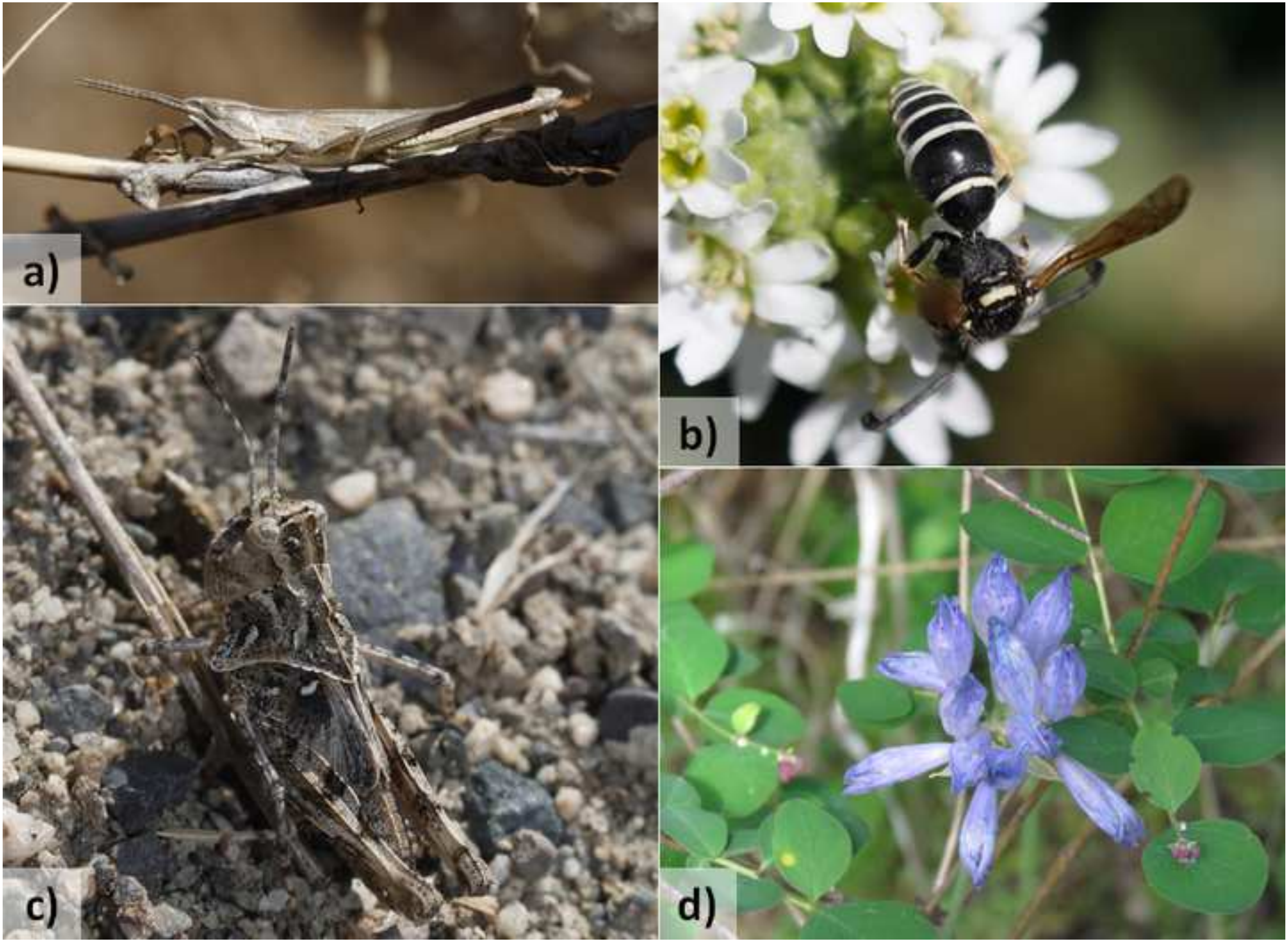

Figure 5

Click here to access/download;Figure;Fig5.tif

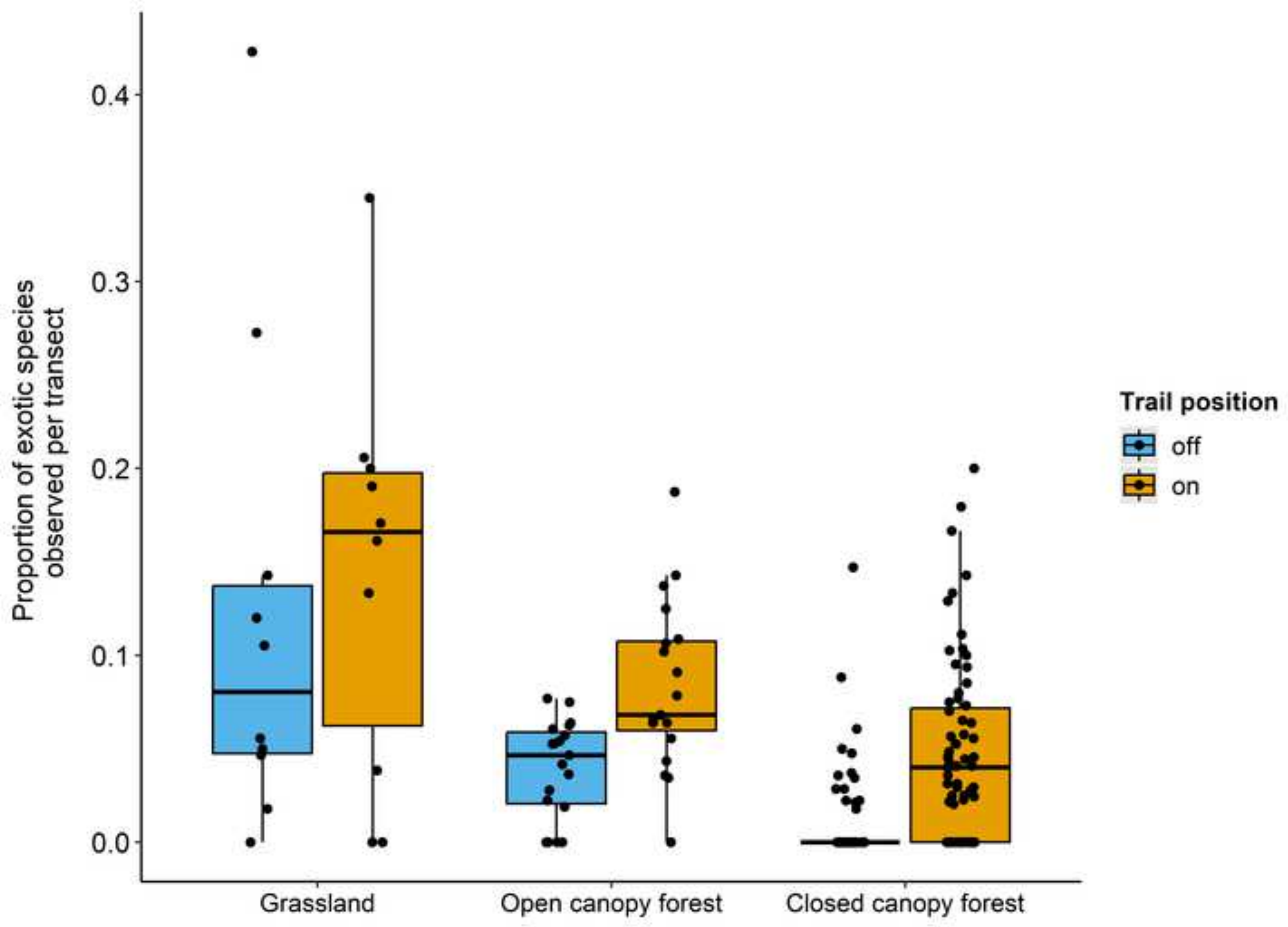

SI Figure 1

Click here to access/download

**Supporting Information**

S1_Fig.tif

SI Table 1

Click here to access/download
**Supporting Information**
S1_Table.pdf
