# Supplementary material for: Not all who wander are lost: Trail bias in community science
Source: PLoS One. 2023 Jun 23;18(6):e0287150. doi: 10.1371/journal.pone.0287150 (PMC10289309; doi:10.1371/journal.pone.0287150)
Supplement: Attachment — Submitted filename: Response_to_Reviewers.docx [file pone.0287150.s005.docx]

Response to reviewers

# Email feedback

## Journal requirements

**1. Please ensure that your manuscript meets PLOS ONE's style requirements, including those for file naming. The PLOS ONE style templates can be found at 
https://journals.plos.org/plosone/s/file?id=wjVg/PLOSOne_formatting_sample_main_body.pdf and** 
[**https://journals.plos.org/plosone/s/file?id=ba62/PLOSOne_formatting_sample_title_authors_affiliations.pdf**](https://journals.plos.org/plosone/s/file?id=ba62/PLOSOne_formatting_sample_title_authors_affiliations.pdf)

Completed

**2. In your Methods section, please provide additional information regarding the permits you obtained for the work. Please ensure you have included the full name of the authority that approved the field site access and, if no permits were required, a brief statement explaining why.**

No permits were required as we did not collect any specimens during our field study.

We added a sentence on lines 181-182 stating no permit were required for this fieldwork.

“No permits were required to conduct this field study in the provincial parks. No specimens were collected. “

**3. We note that Figures 1 and 4 in your submission contain copyrighted images. All PLOS content is published under the Creative Commons Attribution License (CC BY 4.0), which means that the manuscript, images, and Supporting Information files will be freely available online, and any third party is permitted to access, download, copy, distribute, and use these materials in any way, even commercially, with proper attribution. For more information, see our copyright guidelines: http://journals.plos.org/plosone/s/licenses-and-copyright.
 
We require you to either (a) present written permission from the copyright holder to publish these figures specifically under the CC BY 4.0 license, or (b) remove the figures from your submission:
 
a. You may seek permission from the original copyright holder of Figures 1 and 4 to publish the content specifically under the CC BY 4.0 license. 
 
We recommend that you contact the original copyright holder with the Content Permission Form (http://journals.plos.org/plosone/s/file?id=7c09/content-permission-form.pdf) and the following text:
“I request permission for the open-access journal PLOS ONE to publish XXX under the Creative Commons Attribution License (CCAL) CC BY 4.0 (http://creativecommons.org/licenses/by/4.0/). Please be aware that this license allows unrestricted use and distribution, even commercially, by third parties. Please reply and provide explicit written permission to publish XXX under a CC BY license and complete the attached form.”
 
Please upload the completed Content Permission Form or other proof of granted permissions as an "Other" file with your submission. 
 
In the figure caption of the copyrighted figure, please include the following text: “Reprinted from [ref] under a CC BY license, with permission from [name of publisher], original copyright [original copyright year].”
 
b. If you are unable to obtain permission from the original copyright holder to publish these figures under the CC BY 4.0 license or if the copyright holder’s requirements are incompatible with the CC BY 4.0 license, please either i) remove the figure or ii) supply a replacement figure that complies with the CC BY 4.0 license. Please check copyright information on all replacement figures and update the figure caption with source information. If applicable, please specify in the figure caption text when a figure is similar but not identical to the original image and is therefore for illustrative purposes only.**

We received written permission from Kate McKeown, Lena Dietz Chiasson, and Erin Springinotic.

We added “Reprinted from iNaturalist under a CC BY license, with permission from Lena Dietz Chiasson and Erin Springinotic, original copyright 2021.” to Figure 4 on lines 277-278.

Note Kate McKeown took habitat photos as part of the field study protocol and sent all the photos to Ellyne Geurts after the field season for referral and data analysis. Kate did not publish these photos publicly; therefore we did not put the exact text of “Reprinted from [ref] under a CC BY license, with permission from [name of publisher], original copyright [original copyright year]” for Figure 1. Instead we said “Published with permission from Kate McKeown, original copyright 2021.” on lines 102-103.

**4. Please review your reference list to ensure that it is complete and correct. If you have cited papers that have been retracted, please include the rationale for doing so in the manuscript text, or remove these references and replace them with relevant current references. Any changes to the reference list should be mentioned in the rebuttal letter that accompanies your revised manuscript. If you need to cite a retracted article, indicate the article’s retracted status in the References list and also include a citation and full reference for the retraction notice.**

References have been checked and no references have been removed. We added all references requested by the reviewers.

## Additional editor comments

Dear Dr Ellyne Geurts,

Thank you for submitting your study about citizen science and trial bias to PLOS ONE.
Your submission has been evaluated by two reviewers, that suggested "Acceptance After Minor Revision".

Both reviewers consider that your manuscript is interesting, important and well-written. I agree with them. Both reviewers were constructive and provided suggestions to improve the quality of your work. Thus, I ask that you try to follow their suggestions, and my own corrections/suggestions provided below.

Reviewer 1 made several suggestions, and provided some references to improve your text.
You can add those that you consider appropriate.
This reviewer is mainly concerned with some questions in the methods, that you can easily improve/fix.

Reviewer 2 also made several suggestions, and provided some references. Your can insert those that you consider suitable for your text. This reviewer is mainly concerned with the sample based on the effort of only "six observers". I ask that your research team read his/her comments carefully to better deal with this question regarding representativeness (six in thousands of observers). Maybe you can mention the number of observers in your studied region (substantially smaller than all iNaturalist observers). This reduced number of (six) observers would not be a big problem, but the reviewer´s concerns deserve attention, as there is research showing that observer´s characteristics influence the set of species documented by photographs. My suggestion is that you add some sentences in a separate paragraph of the Discussion to talk about this. Maybe, in a short section called "Shortcomings". It would be better if YOU deal with this in the Discussion, than if others write less soft criticism in the future.

Both reviewers also made several suggestions, questions and corrections on the manuscript (yellow marks). Please try to follow those that you consider as appropriate.

## Overall Response to editor

We have examined and addressed all the suggested revisions by the reviewers and editor outlined in the email in this response letter. We also examined the two pdfs with the reviewers’ comments and grammatical edits. We incorporated their edits as much as possible and responded to their comments in this response letter at the end.

We have uploaded a “track changes” and a “clean” copy of the revised manuscript. The line numbers in our responses refer to the “track changes” version.

We appreciate the reviewers’ thorough reading of our manuscript and thoughtful comments and edits. Their contributions and revisions greatly improved the manuscript. We addressed all their concerns and responded individually to each point below.

Additional comments and corrections made by the editor:

**Methods, by the end of page 5, line 100. You have to inform more clearly what types of organisms were included in the surveys. You talk about species richness, exotic, native, but you do not explain what organisms were studied (invertebrates, plants, vertebrates). You need an early sentence in the methods to clarify this.**

We added sentences explaining taxonomic richness on lines 105-109:

“Taxonomic richness includes all taxonomic groups (e.g., plants, animals, fungi). Unique taxa are different organisms observed that may not be identified to species-level. For example, dandelion (*Taraxacum* sp.), darkling beetle (Tenebrionidae), and a lichen (Lecanoromycetes) would be counted as three taxa.”

**Field experiments (line 128 ahead). How long were transects I could not find this information. Where is it ? Please clarify this aspect of transects.** 

We provided the mean and standard of the distances traveled between on and off trail on lines 230-231.

**Through the whole text. Make sure that you use "observation" with the same meaning. It appears (but not really clear) that you considered "observation" as a photograph of a species that could be identified. Was it ? If so, please try to make a sentence for this. It is vague.**

We use the term observation in the context of iNaturalist, which can include photographed or audio recordings of a wild organism that has a date and time. We discuss what makes a verifiable observation in the Methods on lines 127-128.

We have added a small definition on lines 54-55 when the word observation first appears in the introduction.

“iNaturalist engages the largest number of people globally of any biodiversity community science platform, with more than 138 million observations **– geotagged photographed or audio-recorded species records –** of taxa around the world, serving as a huge voucher photo and audio data repository [3].”

Please fix some problems in the References Section:

**Ref 1. Title of the study/book: The initials of all words should not be in italics, except for the first word and names of regions, people, etc.
Please check this for all references.**

We only used italics for the scientific names in the references.

**Refs 3, 5 , others. For online articles, you should use "Available from:". You did not use "from". You did this mistake several times. Please fix.**

We fixed refs 3 (line 388), 5 (line 395), 54 (line 543), 55 (line 545), 61 (line 562),
62 (line 564), 63 (line 566), 64 (line 568), 65 (lines 570), 66 (line 573), and 67 (line 575).

**Ref 6. It should not have a space between initials of first and middle names. Also, delete the dot after initials. Please check this for all references.**

We appreciate the reviewer catching this mistake. It has been corrected for ref 6. We checked all other references and no others were found.

**Refs 7, 8, others. Some titles are divided into two parts, by using ":". When this occur, the first letter should not be in capital. Thus, use ": a case study" instead of ": A case study". It happened several times. Please check.**

Checked and corrected.

**Ref 36. The scientific name of the species should be in italics. Please check all refs.**

Refs 17 and 45 (previously was 36) have been corrected.

5. Review Comments to the Author

**Please use the space provided to explain your answers to the questions above. You may also include additional comments for the author, including concerns about dual publication, research ethics, or publication ethics. (Please upload your review as an attachment if it exceeds 20,000 characters)**

**Reviewer #1: The manuscript is well written and interesting. I have made some comments in the attached pdf. My major comment was trying to incorporate some results of a recently published ms (https://doi.org/10.1111/ibi.13169), with particular attention to their supplementary table about detection differences. I also suggested some other publications in the comments, marked some missing commas with yellow and suggested some clarifications in the methods.**

We appreciate having these papers brought to our attention and the time taken to help improve the manuscript.

We have incorporated the suggested manuscript into our discussion on lines 319-322 and responded to each of the comments down below.

**Reviewer #2: The manuscript by Geurts et al provides an interesting bias evaluation on fine-scale of the contribution of citizen scientists on and off trails in Parks of British Columbia. The results shows that the public can provide a rich contribution observing the biodiversity without leaving the trails, a good perspective for the use of citizen-collected data. The text is well written and I only recommended some references to be cited in the introduction and some minor changes to improve the hole picture. See the pdf attached.**

We appreciate these kind words and the additional literature to enrich the introduction of the manuscript. We have added the suggested references.

**Actually, there is only one thing of my concern, which is related to the choice of using super-observers (only six) to run the field experiments. In fact, it is presumable that these observers are only a particular small subgroup representing volunteers of iNaturalist, because they likely have a superior capacity of organisms’ detection than most of other less productive observers. They are, at least, more experienced than most of other observers in the region to capture images from organisms and submit it to iNaturalist. It is true that the contribution of super-observers covers a large part of the dataset, but summed, the observations of most occasional observers use to be even larger. Other important issue is that they are only six observers. I know that it is a bit annoying, but volunteers may have different behaviors for surveying, often diverging their searching image, experience,** **taxonomic interest, and preferences (see: Bowler et al. 2022: https://doi.org/10.1038/s41598-022-15218-2; Boakes et al. 2016: https://doi.org/10.1038/srep33051; and Rosenblatt et al. 2022:** [**https://doi.org/10.1093/ornithapp/duac008**](https://doi.org/10.1093/ornithapp/duac008)**).**

**For example, are these six observers somehow covering the main observers’ profile in the platform? Maybe it is not enough to cover either the variability inside the 43,690 observers from British Columbia. Considering that “the six” may not represent the most users of iNaturalist, in my opinion, this limitation should be highlighted in the text. Note that this issue does not decrease the relevance of the results and the quality of the contribution, but the inference deserves an adjustment.

Finally, I want to congratulate the authors for the manuscript and I hope to see this paper in the pages of PLOS ONE.**

We realized we were not clear in the field experiments descriptions. We had six team members, but two of them were facilitators for the study and the other four were the observers for the study. Though the whole team were experienced iNaturalist users. This has been corrected in the methods to four observers on line 138.

We have added a paragraph discussing the drawbacks of this facet of the experimental design on lines 343-352.

The focus of the study was to evaluate whether fine-scale spatial bias affects biodiversity measurements, and we used observers that would best allow us to answer this question. We recognize that our study does not consider all variability that iNaturalist observers exhibit, but we selected observers based on the best-case scenario.

# Responses to comments in pdf “PONE-D-23-09371_reviewed”

*Please note the line numbers for the reviewer references the original manuscript. Our responses reference the line numbers in the revised manuscript with track changes.*

**The manuscript is well written and interesting. I have made some comments in the attached pdf. My major comment was trying to incorporate some results of a recently published ms (Edwards et al 2023** [**https://doi.org/10.1111/ibi.13169**](https://doi.org/10.1111/ibi.13169)**), with particular attention to their supplementary table about detection differences. I also suggested some other publications in the comments, just for your interest that could enrich your discussion. I have marked some missing commas with yellow and suggested some clarifications in the methods.**

We have added Edwards et al. 2023 in our discussion on lines 319-322 as support that detectability is likely varying between on and off trail for iNaturalist observers.

We added all commas highlighted and included all clarification edits suggested such as rearranging sentences and simplifying information. We responded to the comments below.

**Line 56: “therefore it can be considered unstructured surveying”**

We agree that iNaturalist data is considered unstructured, however, since the focus of the sentence is on the users, not the study design and data type, we decided not to add this edit on line 56. We instead mention it in the discussion on line 308.

**Line 56: “... at any time”**

Added on line 59.

**Line 64: “you might find useful Tulloch and Szabo. 2012. "A behavioural ecology approach to understand volunteer surveying for citizen science datasets." Emu 112: 313–325”**

We added another point to include habitat preference and reference Tulloch and Szabo 2012 on line 67.

**Line 74: “what is the definition of experienced? with all taxa?”**

It is referring to the experience (number of years) the observers had with the iNaturalist platform and conducting general biodiversity surveys with the platform. We added this information on line 78:

“…2-5 years using the platform…”

to clarify what we mean by experienced.

**Line 82: “is this the official IUCN category? confusing what you mean by rare and vulnerable, they need to be defined” and “after reading the methods, it is better to define it as locally threatened”**

We have replaced all mentions of “vulnerable” with “locally threatened” in the manuscript.

**Line 100: “not sure if i got it right. if you have one bird identified as passer domesticus and another as passer (genus only) do they count as 2 taxa?”**

Yes, that is correct, the above example would count as two taxa. We were not able to use the iNaturalist algorithm to count the number of “leaf” taxa, which would mean in this example it would count as one taxon *i.e.* one “leaf species”. There was the option to restrict to just species-level observations, but we would lose a lot of information as many of the arthropods and lichens could not be identified to species.

We knew there would be some double counting with this method, but we had no reason to think that double counting will happen more frequently on trail than off trail or vice-versa.

To clarify this section, we added the following information on lines 105-109:

“Taxonomic richness includes all taxonomic groups (e.g., plants, animals, fungi). Unique taxa are different organisms observed that may not be identified to species-level. For example, dandelion (*Taraxacum* sp.), darkling beetle (Tenebrionidae), and a lichen (Lecanoromycetes) would be counted as three taxa.”

**Line 175: “I think it would help to italicise variable names throughout the text”**

We did not implement this suggestion. We already italicize the species names and R package names. Adding another group of italicize words would be confusing to the reader.

**Line 201: “i think it would be more helpful to detail what you did with each package, similar to where you mention lme4”**

Many of packages we used were for data cleaning, management, and visualization. So, we added additional information on lines 214-216.

“We conducted the statistical analyses **and data preparation** in R (version 4.1.2.) and RStudio [61,62]. The packages we used **for statistical analyses was** lme4 [60], **and for data cleaning, management, and visualization we used** lubridate [63], dplyr [64], bbmle [65], sp [66], rgdal [67], adehabitatLT [68], and ggplot2 [69].”

**Line 221:”** *** indicates p<0.05”**

Implemented.

**Line 268: “i dont think it needs to be table, can be included as one or two sentences in the results range (mean) on vs off.”**

We appreciate the feedback, but with 10 numerical values in the table, we feel it would be more readily understandable using a table.

**Line 291: “please see Edward et al 2023. "Point count offsets for estimating population sizes of north American landbirds." Ibis 165: 482–503.** [**https://doi.org/10.1111/ibi.13169**](https://doi.org/10.1111/ibi.13169)**.”**

We thought the paper fits better in the next paragraph where we can discuss species detection. We added on lines 319-322:

“Detectability differences between roadside and offroad surveys have been documented for birds, with surveys on roads having greater detectability of bird species when in high forest coverage s regions, and the opposite effect in forest coverage is low (Edwards et al. 2023)”

**Line 299: “just a note - people also make less noise wading through the habitat, thereby not scaring away birds, mammals, reptiles”**

That’s a great point. We added a short note on line 318:

“However, species may also have been more readily detected from trails due to increased visibility, **less noise disturbance**, and greater ease of observers moving through the habitat.”

**Line 310: was this mentioned in the results? if not, i suggest also spelling it out there**

Yes, we have mentioned these results on lines 248-250 in the results. It is also shown in Table 1 and Figure 2.

**Line 325: “see Tulloch et al 2013. "To boldly go where no volunteer has gone before: predicting volunteer activity to prioritize surveys at the landscape scale." Diversity and Distributions 19: 465–480.”**

Added the reference on line 359.

# Responses to comments in pdf “PONE-D-23-09371_reviewer”

*Please note the line numbers for the reviewer references the original manuscript. Our responses reference the line numbers in the revised manuscript with track changes.*

**Line 44: “you should cite also for habitat use, see:**

**Deshwal et al. (2021). Using long-term citizen science data to understand distribution and habitat use of an irruptive species. Ecological Informatics, 64, 101377.**

**Forti et al. (2022). Data collected by citizen scientists reveal the role of climate and phylogeny on the frequency of shelter types used by frogs across the Americas. Zoology, 155, 126052.”**

We added these papers on line 44.

**Line 46: “and for understanding large scale effects of climate on animals behavior, see:**

**Forti et al. (2022). Climate drives anuran breeding phenology in a continental perspective as revealed by citizen‐collected data. Diversity and Distributions, 28(10), 2094-2109.”**

We added this information on line 47.

**Line 50 “cite these papers:**

**Harlin et. Al. (2018). Turning students into citizen scientists. UCL Press.**

**Callaghan et. Al. (2022). The benefits of contributing to the citizen science platform iNaturalist as an identifier. PloS biology, 20(11), e3001843.**

**Forti, L. R. (2023). Students as citizen scientists: project-based learning through the iNaturalist platform could provide useful biodiversity data. Biodiversity, 1-3.”**

We added these three papers on line 52.

**Line 57: “e.g. Kirchhoff et al. (2021). Rapidly mapping fire effects on biodiversity at a large-scale using citizen science. Science of the Total environment, 755, 142348.”**

Added on line 61.

**Line 67: “addressed”**

We replaced “conducted” with “addressed” as suggested on line 70.

**Line 69: “fill”**

We replaced “address” with “fill” as suggested on line 72.

**Line 78: “I think this sentence belongs to M&M, and is not so relevant here.”**

We removed it from the introduction and placed it in the material and methods on lines 153-154

**Line 79: “is this based on the intermediate disturbance hypothesis? Are there similar papers comparing biodiversity on and off trails?”**

It is based on other standard diversity studies and the intermediate disturbance hypothesis. We predicted this outcome due to the intermediate disturbance hypothesis. We added “based on the intermediate disturbance hypothesis” on line 83 and added another reference to support this prediction.

“Swart RC, Pryke JS, Roets F. The intermediate disturbance hypothesis explains arthropod beta-diversity responses to roads that cut through natural forests. Biol Conserv. 2019;236: 243–251. doi:10.1016/j.biocon.2019.03.045”

**Line 121: “it is, more precisely, a consensus of at least 2/3 of the identifiers.”**

That is the correct definition. However, we have struggled to write the official definition in a clear way for the reader that may be unfamiliar with iNaturalist. We hope that readers that want a more precise definition will consult iNaturalist.

As a result, we left the definition as we believe it will allow more readers to understand the general definition of “Research Grade” observations than the official definition.

**Line 132: “With only a small group (six) of super-observers, the results cannot be inferred to the whole community of observers using iNaturalist. Unfortunately, it is instead very limited to a particular profile, which not represent the most users of iNaturalist. This limitation should be highlighted in the text.”**

We address this concern above on page 7. We have added this consideration to the discussion (lines 343-352).

**Line 135: “therefore, the sample leveled up from unstructured to semi-structured collection, right? Could it impact the conclusion? Most of iNaturalist volunteers are not trained for using a particular search pattern…”**

The observers were not directed to observe more a particular taxa or microhabitat. It was still up to the observer discretion on what species they prioritize to observe. The training was to ensure that people were thoroughly familiar with doing the biodiversity surveys and using the app, including people who were working with us for the first time that summer. We wanted a settling in and training period, so that the new team members would not have a larger improvement in surveying behaviour throughout the season than the veterans, and potentially impose a temporal element to the study that we wanted to minimize.

We do not think that allowing observers to learn from each other before beginning the experiments would impact the conclusions, as the observers after the training still exhibited different observing styles. For example, some observers tended to move relatively quickly to find large showy new species while others would spend time on one tree examining lichens and mosses before moving on. The observers also differed in what camera equipment they used.

As a result, we made no changes in the manuscript regarding this comment.

**Line 209: “best?”**

Updated to “Best” on line 224.

**Line 276: “Still in results could be interesting include a description on the representation of different taxonomic groups among on and off trails.”**

We agree that it would interesting, but it is outside the scope of this study. We also think due to the variety of habitats sampled and variability of taxonomic preferences among the observers that it may not be particularly informative for comparing between on and off trails.

We do have the data we used available on Github and Zenodo if this information were something that another researcher would like to examine.

**Line 279: “this should be softened a bit, because the conclusion is based on the behavior of only six super-observers (a specific subgroup of community scientists)”**

That is a good point, but we want the first paragraph to highlight the main results, so we instead discuss our interpretation and survey design in more detail later in the discussion on lines 343-352.

In addition, we think if anything, that with more experienced observers that know how to bushwhack, meander to good microhabitats, and notice things that are obscured by vegetation that it resulted in a small (significant) difference in the number of species recorded between on and off trails than if we had used more inexperienced observers, where we would have expected a much larger difference in species richness between on and off trail.

**Line 290: “I am not sure if it is so "typical". The experiment was carried out using six observers with a presumed higher profile for organisms detection than most of the users of iNat in British Columbia.”**

We did not intend to imply that the observers in our survey represent the common iNaturalist user, but instead reflect an aspect of the iNaturalist community.

We wanted to draw attention that this study did not use fixed area surveys (*e.g.* quadrats and fixed-length transects) that is standard in other diversity studies, but instead the observers used an unstructured community science platform to collect biodiversity data.

We updated the sentence on lines 306-308 to instead say:

“Our test for effects of trails on estimates of taxonomic richness is novel because it includes all taxa in a variety of habitats in time-controlled transects, where observers conducted unstructured community science surveys.”
